# Atlanta Academy of Medicine

**Published:** 1879-06

**Authors:** 


### ATLANTA ACADEMY OF MEDICINE.

ATLANTA, May 10th, 1879.

The Academy was ca'led to order by Dr. J. G. Westmoreland, President *pro tem.*

Dr. Wilson was called to see Mr. G., aged 55, about nine days ago; informed by the family that he had been unwell for two months past; had been suffering with indigestion and intense pain in frontal region of head and in his eyes, which he imagined to be neuralgia. During this time he was listless and would sleep for hours during the day; would forget the names of some of his best friends, which circumstance first attracted their attention to him. When Dr. W. saw him, he had been deprived of consciousness for a few hours; his pupils were slightly contracted, and did not dilate when shaded; pulse about seventy-eight beats; kidneys in normal condition; bowels constipated. Gave him mercurial cathartic and blister applied from medulla oblongata down whole length of spine. Saw him again next morning, mercurial had not acted but blister had drawn well; again administered mercurial and this acted freely during the day. There was no perceptible improvement consequent on this, but patient began to be very restless, for which I administered pot. bromide with desired result. Quinine and iodide of potash given regularly with hope of eliminating any blood poison which might be present. He was nourished with milk and beef tea which he took whenever offered. In the last three days he has had fever, followed by profuse perspiration; kindneys are as yet active, but there is no improvement, on contrary, he is more prostrated.

---

